# TPHE-Graphene: A First-Principles Study of a New 2D Carbon Allotrope for Hydrogen Storage

**DOI:** 10.1021/acsomega.5c04622

**Published:** 2025-08-06

**Authors:** José A. S. Laranjeira, Nicolas F. Martins, Kleuton Antunes Lopes Lima, Luis A. Cabral, Luiz A. Ribeiro Júnior, Douglas S. Galvão, Julio R. Sambrano

**Affiliations:** † Modeling and Molecular Simulation Group, School of Sciences, 28108 São Paulo State University (UNESP), Bauru 17033-360, SP, Brazil; ‡ Department of Applied Physics and Center for Computational Engineering and Sciences, State University of Campinas, Campinas 13083-859, SP, Brazil; § Department of Physics and Meteorology, School of Sciences, 28108 São Paulo State University (UNESP), Bauru 17033-360, SP, Brazil; ∥ Institute of Physics, 28127 University of Brasília, 70919-970 Brasília, DF, Brazil; ⊥ Computational Materials Laboratory, LCCMat, Institute of Physics, University of Brasília, 70919-970 Brasília, DF, Brazil

## Abstract

The shift from fossil fuels to renewable energy sources is essential for reducing global carbon emissions and addressing climate change. Developing advanced materials for efficient hydrogen storage enables the development of sustainable energy solutions in this context. Herein, we propose sodium-decorated TPHE-graphene as a high-performance two-dimensional material for hydrogen storage. Density functional theory calculations and molecular dynamics simulations demonstrate that TPHE-graphene exhibits dynamical, thermal, energetic, and mechanical stability. The monolayer displays metallic behavior and a high Young’s modulus of 250.46 N/m. Upon sodium decoration, strong chemisorption occurs with a binding energy of −2.08 eV and minimal tendency for Na atom clustering. Hydrogen adsorption analysis reveals that each Na atom can bind up to five H_2_ molecules, resulting in a gravimetric storage capacity of 9.52 wt %. The calculated H_2_ adsorption energies range from −0.22 to −0.18 eV, falling within the ideal range for reversible adsorption under ambient conditions. These findings highlight Na-decorated TPHE-graphene as a structurally robust and efficient hydrogen storage material well-suited for future green energy applications.

## Graphical abstract

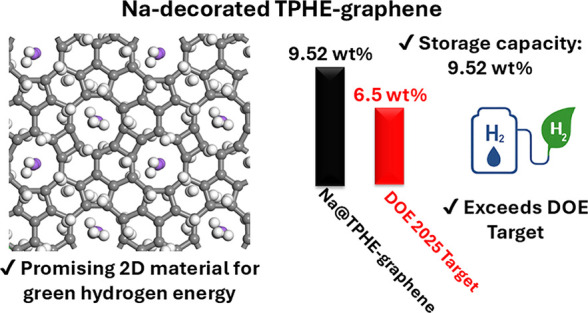

## Introduction

Transitioning from traditional fossil fuels to renewable energy sources is essential for reducing global carbon emissions and mitigating associated environmental impacts and health issues.^1^ In this context, hydrogen (H_2_) stands out as an environmentally friendly energy carrier capable of meeting the demands of modern technologies.^2,3^ Unlike other clean energy sources such as solar and wind, hydrogen-based energy systems are not weather-dependent. However, a significant challenge lies in the efficient storage of H_2_, given its low volumetric and gravimetric energy densities. Depending on the storage method, hydrogen often needs to be stored under high-pressure conditions.^4,5^

Since the seminal report by Reilly and Wiswall^6^ demonstrating the excellent hydrogen storage performance of Mg_2_NiH_4_, solid-state hydrogen storage has gained significant attention.^7^ Despite continued exploration of various platforms such as nanotubes,^8,9^ metal–organic frameworks (MOFs),^10,11^ and metal hydrides,^12^ many of these materials suffer from low gravimetric hydrogen capacity, primarily due to their high molecular weights. To address this limitation, researchers have proposed tuning the nanostructure dimensionality of host materials to improve hydrogen uptake.^13^

Two-dimensional (2D) materials, in particular, have emerged as promising candidates for sustainable energy applications, owing to their high surface-area-to-weight ratio and ultralight atomic structures.^14–16^ Graphene-like materials exemplify these characteristics. However, the intrinsically low reactivity of pristine carbon networks leads to weak interactions with hydrogen molecules, resulting in limited adsorption efficiency.^17^

A common strategy to enhance the interaction between hydrogen molecules and two-dimensional (2D) carbon-based materials is the use of metal decoration. In particular, alkali earth metals (Be, Mg, Ca) and alkali metals (Li, Na, K) offer a promising approach due to their low atomic masses, which align with the criteria for high gravimetric hydrogen capacity.^18,19^ Moreover, metal decoration typically induces a physisorption regime characterized by optimal adsorption energies of −0.10 to −0.40 eV,^20^ suitable for reversible hydrogen uptake under ambient conditions.

During the past decade, considerable effort has been dedicated to exploring novel 2D carbon allotropes for hydrogen storage applications.^21–23^ The recently synthesized biphenylene sheet has demonstrated excellent performance in retaining H_2_ molecules.^24,25^ First-principles calculations have further confirmed the high hydrogen adsorption capacities in various functionalized 2D carbon materials, including irida-graphene,^26,27^ PAI-graphene,^28^ PHE-graphene,^29^ holey-graphyne^30^ and M-graphene.^31^

Recently, Shi et al.^32^ employed a random structure search strategy combined with graph theory to explore novel two-dimensional (2D) *sp* ^2^ carbon allotropes. Their study identified 1114 previously unreported carbon structures, among which 190 were classified as Dirac semimetals, 241 as semiconductors, and 683 as conventional metals. These materials exhibit various exotic electronic behaviors, including type III, type I/II mixed, and type I/III mixed semimetallic characteristics.

From this extensive data set, we focus on one particular structure, TPHE-graphene, among the configurations proposed in their work. TPHE-graphene presents a unique 2D lattice composed of square, pentagonal, hexagonal, and enneagonal carbon rings arranged in a rectangular geometry and belonging to the *Pmma* space group (No. 51). Our particular interest is the spatial distribution of its enneagonal rings, which are well-separated and symmetrically positioned—creating an ideal topology for anchoring metal adatoms and, consequently, enhancing hydrogen (H_2_) adsorption through decoration strategies.

In response to the growing demand for novel 2D materials that surpass the minimum requirements for hydrogen (H_2_) storage capacity, we propose sodium-decorated TPHE-graphene as a promising candidate, based on first-principles calculations within the density functional theory (DFT) framework. Sodium (Na) was selected for decoration due to its low atomic mass, high reactivity, and low melting point, contributing to enhanced interactions with H_2_ molecules.

Herein, we systematically investigate the hydrogen adsorption behavior of Na-decorated TPHE-graphene through a comprehensive set of computational analyses, including molecular dynamics simulations, charge density difference (CDD), projected density of states (PDOS), adsorption and consecutive adsorption energy calculations, hydrogen adsorption capacity (HAC), and thermodynamic modeling. Furthermore, we assessed the dynamical, thermal, and mechanical stability of the pristine TPHE-graphene monolayer, providing valuable insights into its viability as a high-performance hydrogen storage substrate. According to the U.S. Department of Energy (DOE), a viable hydrogen storage system for light-duty vehicles must achieve a gravimetric capacity of at least 5.5 wt % (ultimate target: 6.5 wt %), with reversible adsorption and desorption occurring near ambient temperature and pressure.^33^ Our results demonstrate that Na-decorated TPHE-graphene exceeds these gravimetric targets, while maintaining physisorption energies within the desirable range for practical use.

## Methodology

First-principles calculations based on DFT were performed to investigate the structural and hydrogen storage properties of TPHE-graphene. Exchange-correlation effects were treated using the generalized gradient approximation (GGA) as proposed by Perdew, Burke, and Ernzerhof (PBE).^34,35^ The projector-augmented wave (PAW) method^36^ was used for the interaction between the core and the valence electrons. All simulations were performed using the Vienna ab initio Simulation Package (VASP).^37,38^ The set of plane-wave basis was defined with an energy cutoff of 520 eV. To avoid spurious interactions due to periodic boundary conditions, a vacuum layer of 15 Å was added along the *z*-direction.

Structural optimization and projected density of states (PDOS) calculations were carried out using 6 × 6 × 1 and 9 × 9 × 1 **k**-point meshes centered at the Γ-point, respectively. The van der Waals interactions were taken into account using the Grimme DFT-D2 method.^39^ Geometry optimization used the conjugate gradient algorithm with convergence thresholds of 10^–5^ eV for total energy and 0.01 eV/Å for atomic forces. Charge transfer analysis was conducted using the Bader partitioning scheme.

Molecular dynamics (MD) simulations were performed using the tight-binding approximation available in the DFTB+ package,^40^ employing the 3ob parametrization.^41^ These MD simulations were performed with the Berendsen thermostat^42^ at 300 K for 5 ps and a time step of 1 fs.

The energetic stability of TPHE-graphene was analyzed by its cohesive energy (*E*_coh_):

$$E_{\mathrm{coh}}=\frac{E_{\mathrm{TPHE}}-\underset{i}{\sum }n_{i}E_{i}}{\underset{i}{\sum }n_{i}}$$ 1

In this formulation, *E*_TPHE_ stands for the total energy of the system, while *E*_*i*_ represents the energy of an isolated carbon atom, and *n*_*i*_ denotes the number of C atoms in the structure. The same procedure was consistently applied to evaluate the cohesive energy (*E*_coh_) of the other 2D materials examined throughout this study.

The charge density difference (CDD) obtained from the Na@TPHE-graphene system was calculated by

$$\Delta \rho =\rho_{\left(\mathrm{Na}@\mathrm{TPHE}\right)}-\rho_{\left(\mathrm{Na}\right)}-\rho_{\left(\mathrm{TPHE}\right)}$$ 2

where ρ_(Na@TPHE)_, ρ_(Na)_, and ρ_(TPHE)_ refer to the charge densities of the Na@TPHE-graphene substrate, isolated Na adatoms, and pristine TPHE-graphene monolayer, respectively. For the hydrogen-adsorbed Na@TPHE-graphene system, the CDD was obtained as

$$\Delta \rho =\rho_{\left(\mathrm{Na}@\mathrm{TPHE}+H_{2}\right)}-\rho_{\left(H_{2}\right)}-\rho_{\left(\mathrm{Na}@\mathrm{TPHE}\right)}$$ 3

where ρ_(Na@TPHE+H_2_)_, ρ_(H_2_)_, and ρ_(Na@TPHE)_ refer to the charge densities of the Na@TPHE-graphene with H_2_ molecules, isolated H_2_ molecules, and Na@TPHE-graphene substrate, respectively.

The adsorption energy (*E*_ads_) for Na@TPHE-graphene + *n*H_2_ systems was computed using the following expression:

$$E_{\mathrm{ads}}=\frac{1}{n}(E_{\mathrm{Na}@\mathrm{TPHE}+nH_{2}}-E_{\mathrm{Na}@\mathrm{TPHE}}-nH_{2})$$ 4

where *E*_Na*@*TPHE+*n*H_2__ represents the total energy of the Na@TPHE-graphene system with *n* adsorbed H_2_ molecules, *E*_Na@TPHE_ is the energy of the bare Na@TPHE-graphene substrate, and *E*_H_2__ corresponds to the energy of an isolated H_2_ molecule.

The consecutive adsorption energy (*E*_con_) for each H_2_ molecules addition on the surface was determined as

$$E_{\mathrm{con}}=\frac{1}{4}(E_{\mathrm{Na}@\mathrm{TPHE}+nH_{2}})-E_{\mathrm{Na}@\mathrm{TPHE}+(n-4)H_{2}}-4E_{H_{2}}$$ 5

The hydrogen adsorption capacity (HAC) in weight percentage was calculated as

$$\mathrm{HAC}(\mathrm{wt}\;\%)=\frac{n_{H}M_{H}}{n_{C}M_{C}+n_{\mathrm{Na}}M_{\mathrm{Na}}+n_{H}M_{H}}$$ 6

where *n*_*X*_ and *M*_*X*_ represent the number of atoms and molar masses of element *X* (X = H, C, Na), respectively.

Assuming atmospheric pressure (1 atm), the hydrogen desorption temperature (*T*_des_) was estimated using the van’t Hoff equation^43,44^ :

$$T_{\mathrm{des}}=\left|E_{\mathrm{ads}}\right|\frac{R}{K_{B}\Delta S}$$ 7

where *R* denotes the universal gas constant, *k*_B_ represents the Boltzmann constant, and Δ*S* corresponds to the change in entropy during the hydrogen phase transition from gas to liquid, taken as 75.44 J mol^–1^ K^–1^.

A thermodynamic analysis was performed to evaluate the adsorption and desorption behavior of H_2_ molecules under realistic conditions, employing the grand canonical partition function *Z*, given by

$$Z=1+\overset{n}{\underset{i=1}{\sum }}\exp\left(-\frac{E_{i}^{\mathrm{ads}}-\mu }{k_{B}T}\right)$$ 8

In this equation, *n* denotes the total number of H_2_ molecules that can be adsorbed, while μ represents the chemical potential of a hydrogen molecule in the gaseous state. Here, *E*_*i*_^ads^ corresponds to the adsorption energy of the *i*-th H_2_ molecule.^24,45^

## Results and Discussion

### Structural, Electronic and Mechanical Features of TPHE-Graphene

As discussed previously, TPHE-graphene features a rectangular lattice that crystallizes in the *Pmma* space group (No. 51). Its atomic structure comprises an arrangement of square, pentagonal, hexagonal, and enneagonal carbon rings, as illustrated in Figure 1a. The optimized lattice parameters were calculated as $\vec{a}$ = 8.75 Å and $\vec{b}$ = 7.79 Å, and the unit cell contains nine nonequivalent atoms.

**Figure 1:**
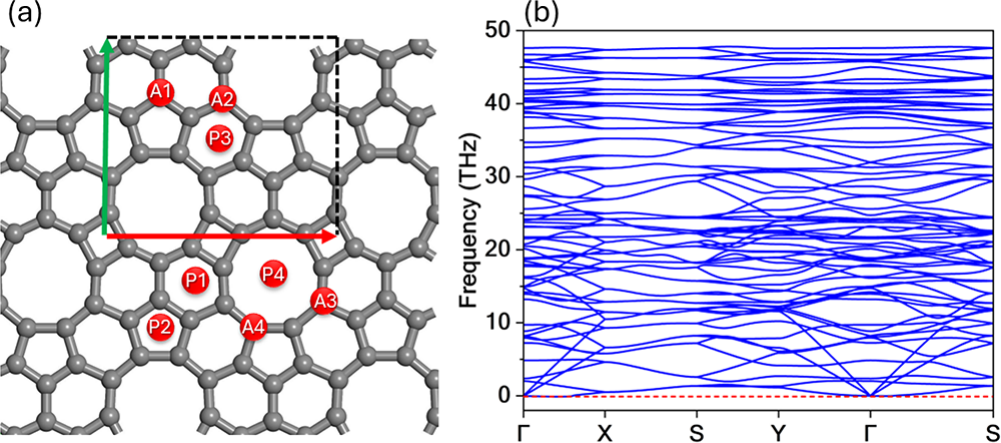
(a) Top view of the TPHE-graphene monolayer highlighting the rectangular unit cell (dashed black lines) and the evaluated high-symmetry adsorption sites for Na decoration. Sites labeled P1–P4 correspond to pore-centered positions, while A1–A4 indicate atomic sites. (b) Phonon dispersion of pristine TPHE-graphene.

To evaluate the energetic stability of TPHE-graphene, we computed its cohesive energy (*E*_coh_), obtaining a value of −7.63 eV/atom. This result is comparable to the cohesive energies of other known 2D carbon allotropes, such as graphene (−7.68 eV/atom), PHE-graphene (−7.56 eV/atom), T-graphene (−7.45 eV/atom), and penta-graphene (−7.13 eV/atom). Based on this comparison with ground-state atomic energies, TPHE-graphene can be classified as energetically stable.

We now examine the phonon dispersion of TPHE-graphene, as presented in Figure 1b. The absence of negative frequencies (i.e., imaginary modes) confirms the dynamical stability of the monolayer. Moreover, the presence of multiple band crossings suggests the existence of several thermal transport channels, which may contribute to enhanced thermal conductivity. The phonon spectrum extends up to 50 THz.

Molecular Dynamics (MD) simulations were performed to further assess the thermal stability of TPHE-graphene. Figure 2 shows the potential energy profile over time (Figure 2a) and the final structure after 5 ps of simulation at 300 K (Figure 2b). The potential energy stabilizes after approximately 0.5 ps, oscillating around a mean value of −1113.2 eV, with fluctuations not exceeding 0.5 eV, as illustrated in Figure 2a. This stability indicates the absence of reconstruction or phase transition events throughout the simulation. Additionally, the final geometry confirms that the structural integrity of the monolayer is preserved, with only minimal distortions observed, most notably a slight buckling in the lattice (see Figure 2b).

**Figure 2:**
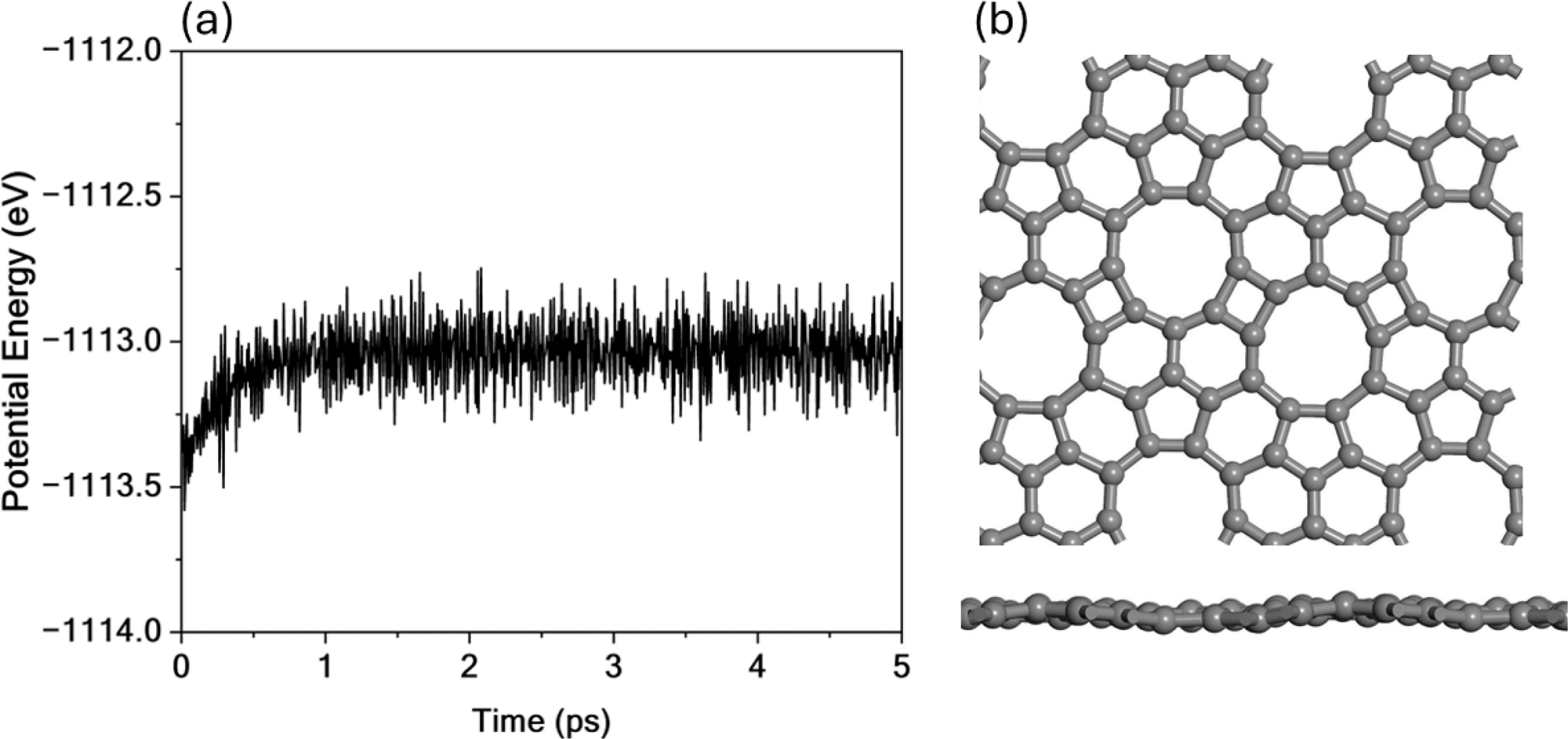
MD simulation results for pristine TPHE-graphene at 300 K. (a) Time evolution of the potential energy over a 5 ps simulation. (b) Top and side views of the final structure.

The electronic structure of TPHE-graphene was investigated through its band structure and PDOS, as shown in Figure 3. The results reveal a metallic character, evidenced by three bands that cross the Fermi level. The highest fully occupied bands exhibit significant dispersion (approximately 2 eV), correlating with a noticeable reduction in the PDOS within that energy range. Near the Fermi level (indicated by the red dashed line), a concentration of partially occupied states accounts for the nonzero PDOS, further supporting the metallic nature of the system.

**Figure 3:**
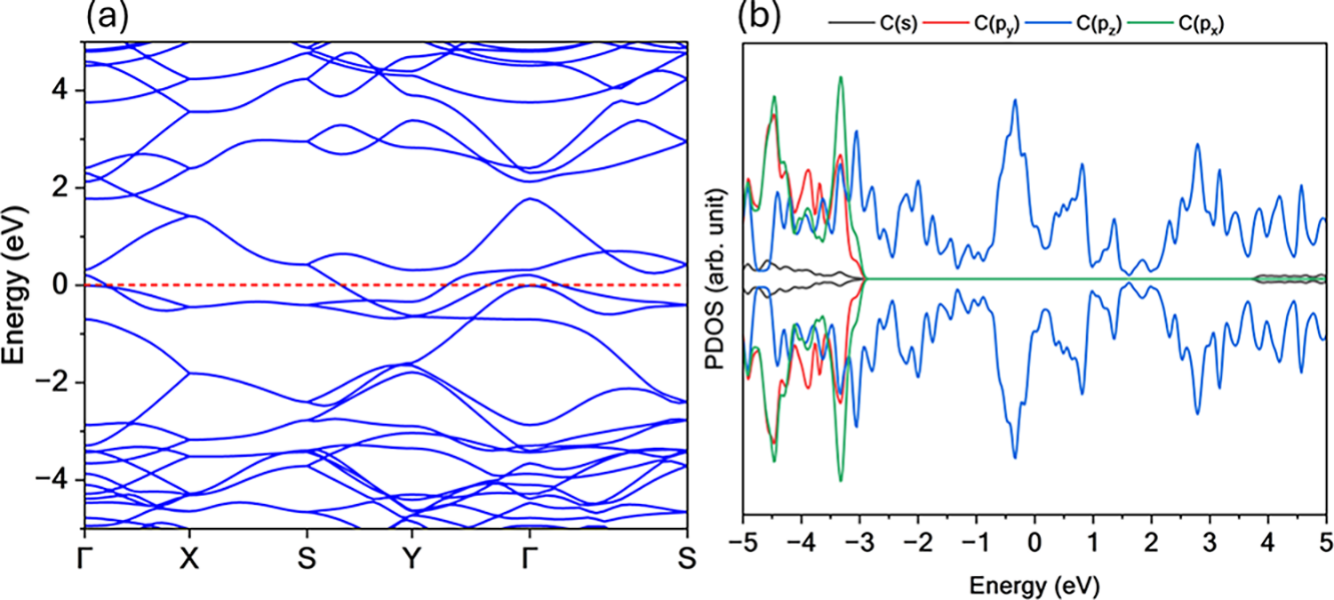
(a) Band structure and (b) PDOS for TPHE-graphene system. This novel monolayer exhibits metallic behavior, characterized by several bands that cross the Fermi level (red dashed line).

Additionally, two tilted cone-like crossings appear along the Γ → *X* and *S* → *Y* directions. Although these features do not exactly align with the Fermi level, they originate from partially filled bands. They are of particular interest due to their potential to host anisotropic charge carriers and support unconventional transport phenomena. In the conduction band, a strong band dispersion is also observed near the Fermi level, accompanied by a corresponding dip in the PDOS, reinforcing the delocalized nature of the electronic states in this region.

The composition of the electronic states was analyzed using PDOS. For lower energies in the valence band, the electronic states are primarily composed of C­(*p*_*x*_) and C­(*p*_*y*_) orbitals, with a minor contribution from C­(*s*) orbitals, indicating the formation of localized σ bonds. For energies above −3 eV, the PDOS is dominated by C­(*p*_*z*_) orbitals, which are associated with the delocalized π system—characteristic of *sp* ^2^-hybridized carbon frameworks. This π-dominance near the Fermi level reinforces the metallic nature of the TPHE-graphene monolayer.

The mechanical properties of TPHE-graphene were evaluated and are illustrated in terms of the Young’s modulus (*Y*), shear modulus (*G*), and Poisson’s ratio (ν) in Figure 4. The Young’s modulus reaches a maximum (minimum) of 250.46 N/m (232.74 N/m), with an anisotropy ratio of 1.08. Similarly, the *G* varies slightly between 94.12 and 90.77 N/m, yielding an anisotropy ratio of 1.04. The Poisson’s ratio exhibits minimal directional dependence, varying from 0.31 to 0.34, with an anisotropy ratio of 1.10. These slight variations indicate that TPHE-graphene behaves as an almost mechanically isotropic material.

**Figure 4:**
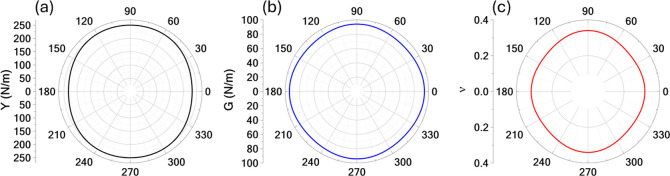
(a) Young’s modulus (*Y*), (b) shear modulus (*G*), and (c) Poisson’s ratio (ν) for TPHE-graphene.

Mechanical stability was further assessed by computing the elastic constants, resulting in *C*_11_ = 260.87 N/m, *C*_22_ = 280.32 N/m, *C*_12_ = 88.80 N/m, and *C*_66_ = 94.13 N/m. For a 2D material with rectangular symmetry, the Born–Huang stability criteria^46,47^ require that *C*_11_ > 0, *C*_66_ > 0, and *C*_11_ *C*_22_ > *C*_12_^2^—all of which are satisfied by TPHE-graphene, confirming its mechanical robustness.

### Sodium Decoration on TPHE-Graphene Monolayer

In this section, we investigate the sodium decoration mechanism on the TPHE-graphene monolayer. Specifically, we evaluated the adsorption of Na atoms at eight high-symmetry sites: P1, P2, P3, and P4, associated with the pores (ring centers) of the lattice, and A1, A2, A3, and A4, which correspond to on-top atomic positions within the carbon framework (refer to Figure 1). The computed adsorption energies and final configurations are summarized in Table 1 and visually represented in Figure 5.

**Table 1:** Adsorption Energies (*E*_ads_) and Final Configurations for the Adsorption Sites Evaluated during Na Decoration on the TPHE-Graphene

initial site	*E*_ads_ (eV)	final site
P1	–1.79	P1
P2	–1.85	P2
P3	–1.92	P3
P4	–2.08	P4
A1	–1.92	P3
A2	–1.79	P1
A3	–1.85	P2
A4	–1.93	P3

**Figure 5:**
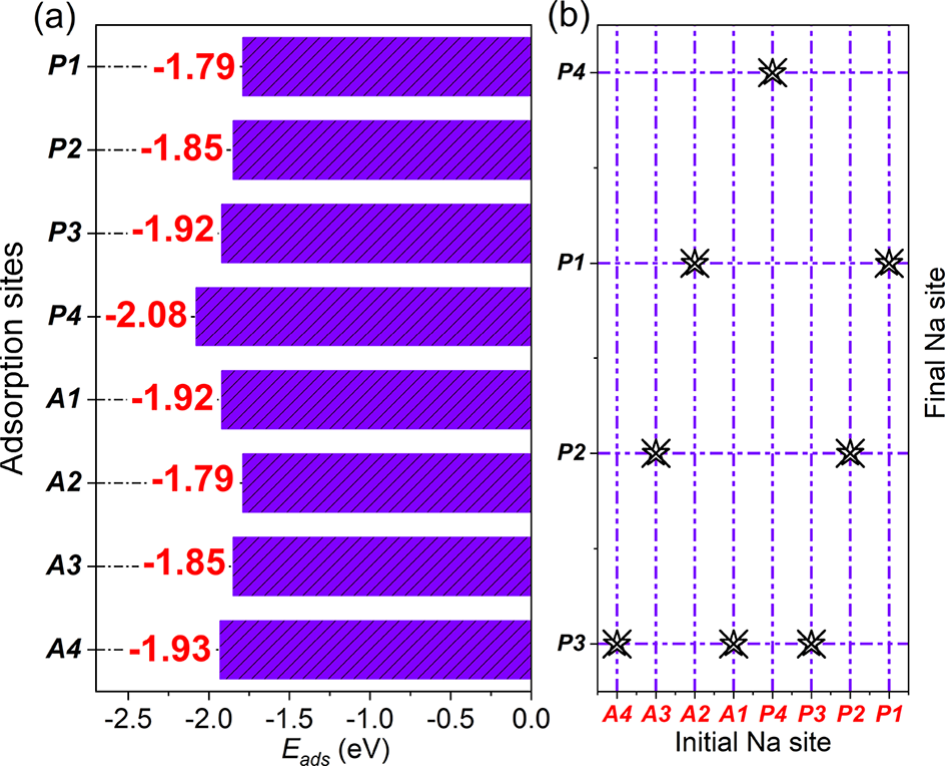
(a,b) Adsorption energies (*E*_ads_) for each adsorption site along with final configurations for the adsorption sites evaluated during Na decoration on the TPHE-graphene. Strong adsorption is observed at the pore sites, with *E*_ads_ values of −1.79, −1.85, and −1.92 eV/atom for the P1, P2, and P3 sites, respectively.

**Table 2:** Bader Charge Analysis for Na@TPHE-Graphene + *n*H_2_ Systems^a^

**system**	**Na (total)**	**H**_**2**_ **(total)**	**TPHE-graphene**
Na@TPHE + 4H_2_	+3.27	–0.21	–3.05
Na@TPHE + 8H_2_	+3.31	–0.30	–3.01
Na@TPHE + 12H_2_	+3.30	–0.34	–2.96
Na@TPHE + 16H_2_	+3.30	–0.37	–2.93
Na@TPHE + 20H_2_	+3.33	–0.33	–2.97

a Values represent the total charge (in |*e*|) for all equivalent atoms or molecules.

Our results indicate that Na adatoms consistently favor adsorption at the pore-centered sites of the TPHE-graphene lattice, irrespective of their initial positions. Among these, the enneagonal pore (P4) emerges as the most favorable adsorption site, exhibiting an adsorption energy (*E*_ads_) of −2.08 eV/atom. This value reflects a strong chemisorption interaction, which promotes the formation of a stable Na@TPHE-graphene complex when the monolayer is fully decorated at the P4 sites. As illustrated in Figure 6, this configuration comprises four Na atoms per TPHE-graphene unit cell.

**Figure 6:**
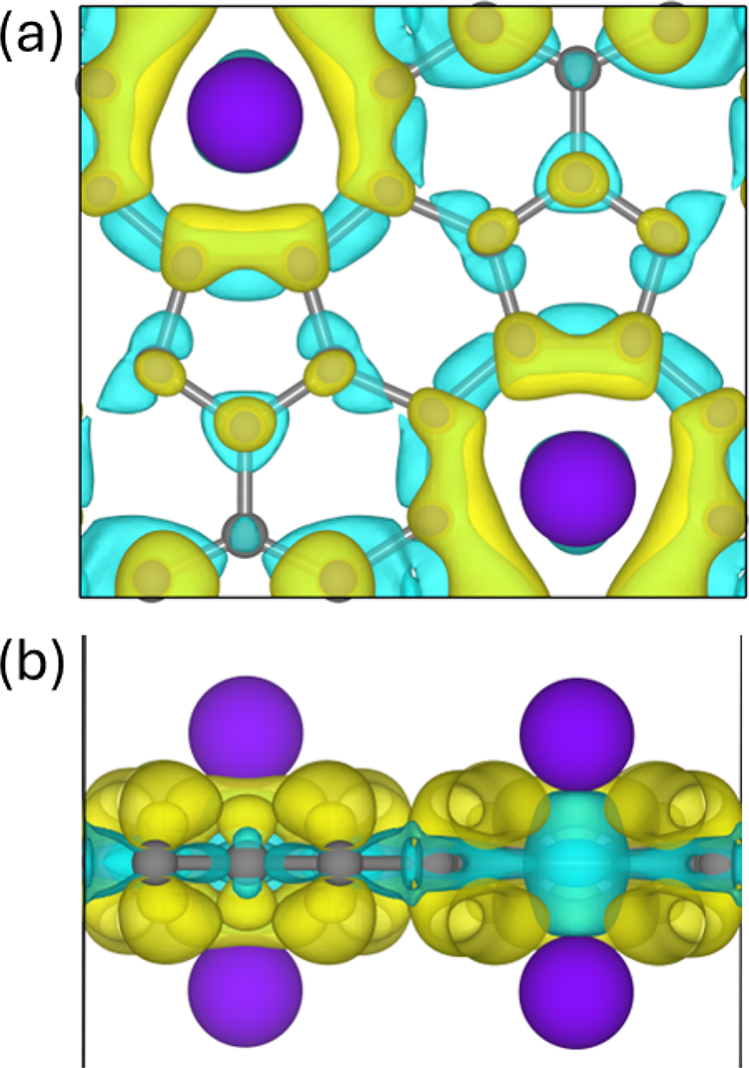
Charge density difference (CDD) visualizations shown from the (a) top and (b) side views for Na@TPHE-graphene system. The yellow (blue) regions indicate charge accumulation (depletion).

Strong adsorption is also observed at the remaining pore sites, with *E*_ads_ values of −1.79, −1.85, and −1.92 eV/atom for the P1, P2, and P3 sites, respectively. These relatively high adsorption energies indicate limited mobility of Na adatoms on the TPHE-graphene surface. Consequently, a high diffusion barrier is anticipated, effectively suppressing adatom clustering and thereby contributing to the structural stability of the Na-decorated monolayer.

To further elucidate the nature of the interaction between Na adatoms and the TPHE-graphene monolayer, we analyzed the charge density difference (CDD) map for the Na@TPHE-graphene system, as depicted in Figure 6. In addition, Bader charge analysis was conducted to quantify the charge transfer resulting from the adsorption process. The CDD map shows pronounced charge accumulation around the enneagonal pores where Na atoms are adsorbed, indicating a net electron transfer from the adatoms to the graphene surface. This observation is corroborated by the Bader analysis, which reveals a charge transfer of approximately −0.75 |*e*| per Na atom to the monolayer. Such substantial charge transfer supports an ionic adsorption mechanism, accounting for the strong binding affinity between Na and the TPHE-graphene structure.

A fundamental requirement for a material to serve as a viable hydrogen storage substrate is its thermal stability under ambient conditions. To assess the thermal stability of Na@TPHE-graphene, MD simulations were conducted at 300 K for 5 ps, as illustrated in Figure 7. In the simulation, the potential energy fluctuates around a stable mean value, with oscillation amplitudes confined to approximately 0.5 eV. This stability suggests that no desorption events or structural reconstructions occurred throughout the simulation. Furthermore, visual inspection of the final structure reveals that the Na atoms remain firmly anchored at their most favorable adsorption sites, exhibiting only minor deviations. These observations collectively confirm the thermal robustness of the Na-decorated TPHE-graphene system at room temperature.

**Figure 7:**
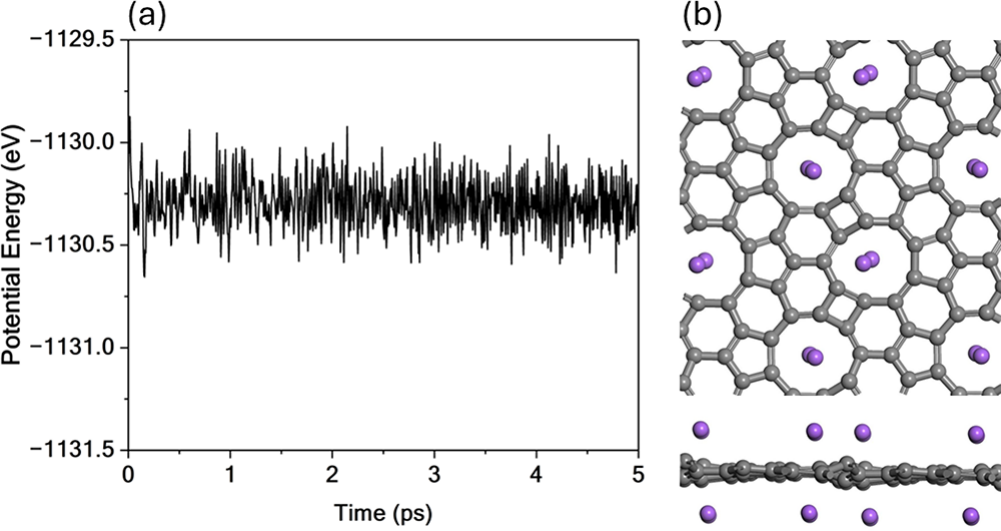
MD simulation results for pristine Na@TPHE-graphene at 300 K. (a) Time evolution of the potential energy and (b) final system configuration for Na@TPHE-graphene.

The surface diffusion of adsorbed Na atoms can lead to clustering, reducing the number of active sites available for hydrogen adsorption. To evaluate the probability of such diffusion, the nudged elastic band (NEB) method^48–50^ was used, as shown in Figure 8. This calculation generated seven images, five of which correspond to transition states.

**Figure 8:**
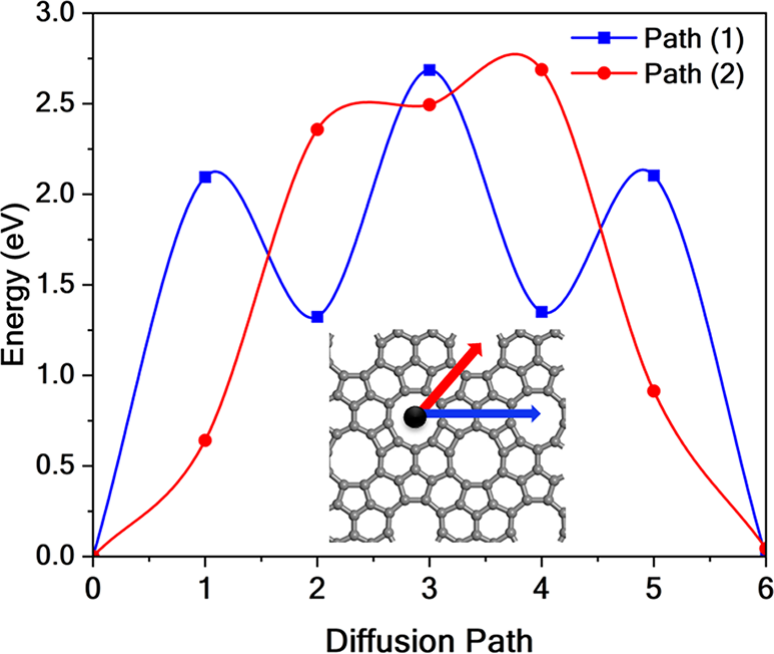
Diffusion energy barrier for Na atom migrates along two paths: the first connecting the enneagonal sites through the naphthylene segments, and the second passing through the distorted hexagonal ring that links adjacent enneagonal pores. The diffusion energy barriers are 2.69 eV for both diffusion paths.

Two diffusion pathways were considered: connecting neighboring enneagonal pores via the naphthylene segments and traversing the distorted hexagonal ring that links adjacent enneagonal sites. The results show that the energy barrier for Na diffusion is approximately 2.69 eV, indicating a low probability of spontaneous diffusion at room temperature. This value is consistent with those reported for similar systems, such as Ca-decorated biphenylene (2.52 eV),^25^ Ti-decorated irida-graphene (5.0 eV),^51^ and Sc-decorated biphenylene (3.48 eV),^52^ further supporting the structural stability of the Na@TPHE-graphene complex.

This energy barrier is significantly higher than the thermal energy available to a single atom at room temperature (300 K), which the classical expression can estimate:

$$E_{\mathrm{thermal}}=\frac{3}{2}k_{B}T$$ 9

where *k*_B_ = 8.617 × 10^–5^ eV/K is the Boltzmann constant. Substituting the temperature:

$$E_{\mathrm{thermal}}=\frac{3}{2}\times 8.617\times 10^{-5}\times 300\approx 0.039\;\mathrm{eV}$$ 10

Therefore, the diffusion barrier of 2.69 eV is almost 70 times greater than the thermal energy at 300 K, indicating that spontaneous diffusion through the pore is thermally inaccessible under ambient conditions and would require elevated temperatures or external activation to occur.

To investigate the impact of sodium adsorption on the electronic structure of TPHE-graphene, we analyzed the band structure and PDOS for the Na@TPHE-graphene system, as shown in Figure 9. The results indicate that the metallic character of the monolayer is preserved, with a single band crossing the Fermi level. The highly dispersive nature of the occupied bands near the Fermi level is also maintained, with a noticeable reduction in the PDOS in this region, suggesting delocalized electronic states.

**Figure 9:**
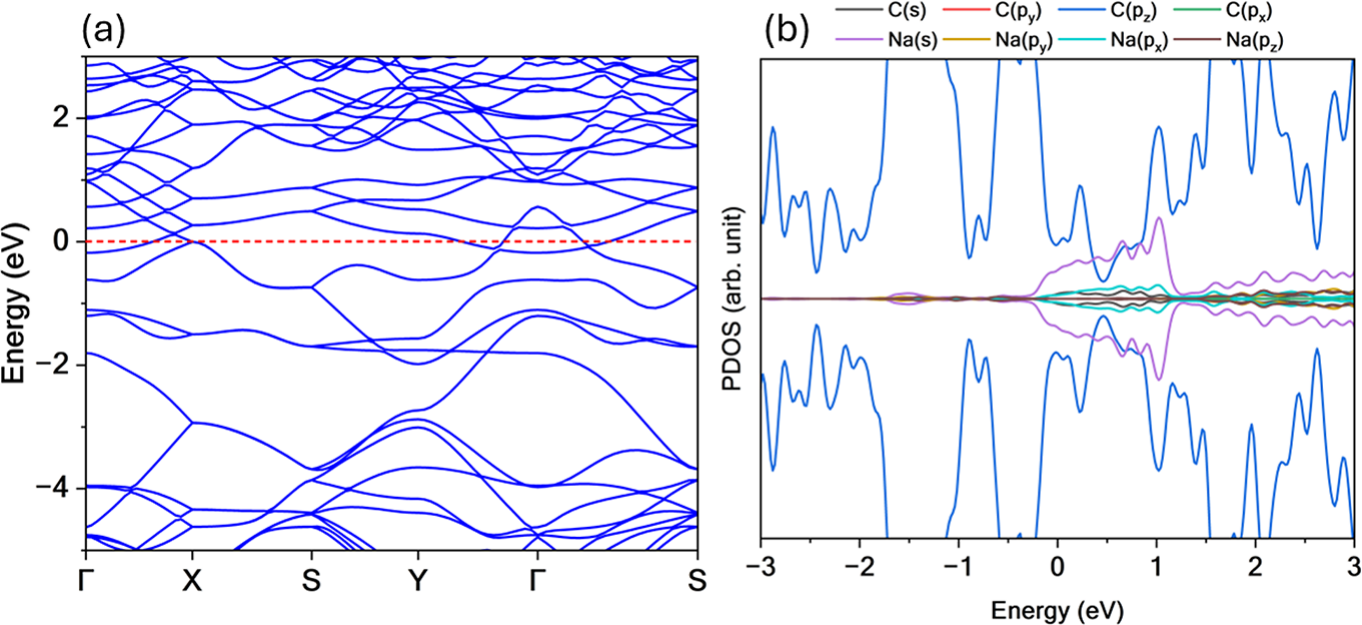
(a) Band structure and (b) PDOS for Na@TPHE-graphene system. The system retains its metallic character with a single band crossing the Fermi level and reduced PDOS in this region.

Several additional bands appear in the conduction band (CB), predominantly associated with Na­(*s*) and Na­(*p*) orbitals, as evidenced in the PDOS. Moreover, tilted cone-like crossings are observed near the Fermi level along the Γ → *X*, *Y* → Γ, and Γ → *S* directions, which may facilitate anisotropic charge transport. Regarding orbital composition, the electronic states are primarily derived from C­(*p*_*z*_) orbitals, with negligible contributions from other carbon states. Notably, Na­(*s*) orbitals contribute significantly near the Fermi level, highlighting the role of sodium in modifying the local electronic environment.

### Hydrogen Storage Properties of TPHE-Graphene

We now evaluate the hydrogen storage potential of the Na@TPHE-graphene system. For this purpose, we sequentially adsorbed one H_2_ molecule per Na adatom, generating a stepwise series of configurations containing 4, 8, 12, 16, and 20 H_2_ molecules per TPHE-graphene unit cell, as illustrated in Figure 10.

**Figure 10:**
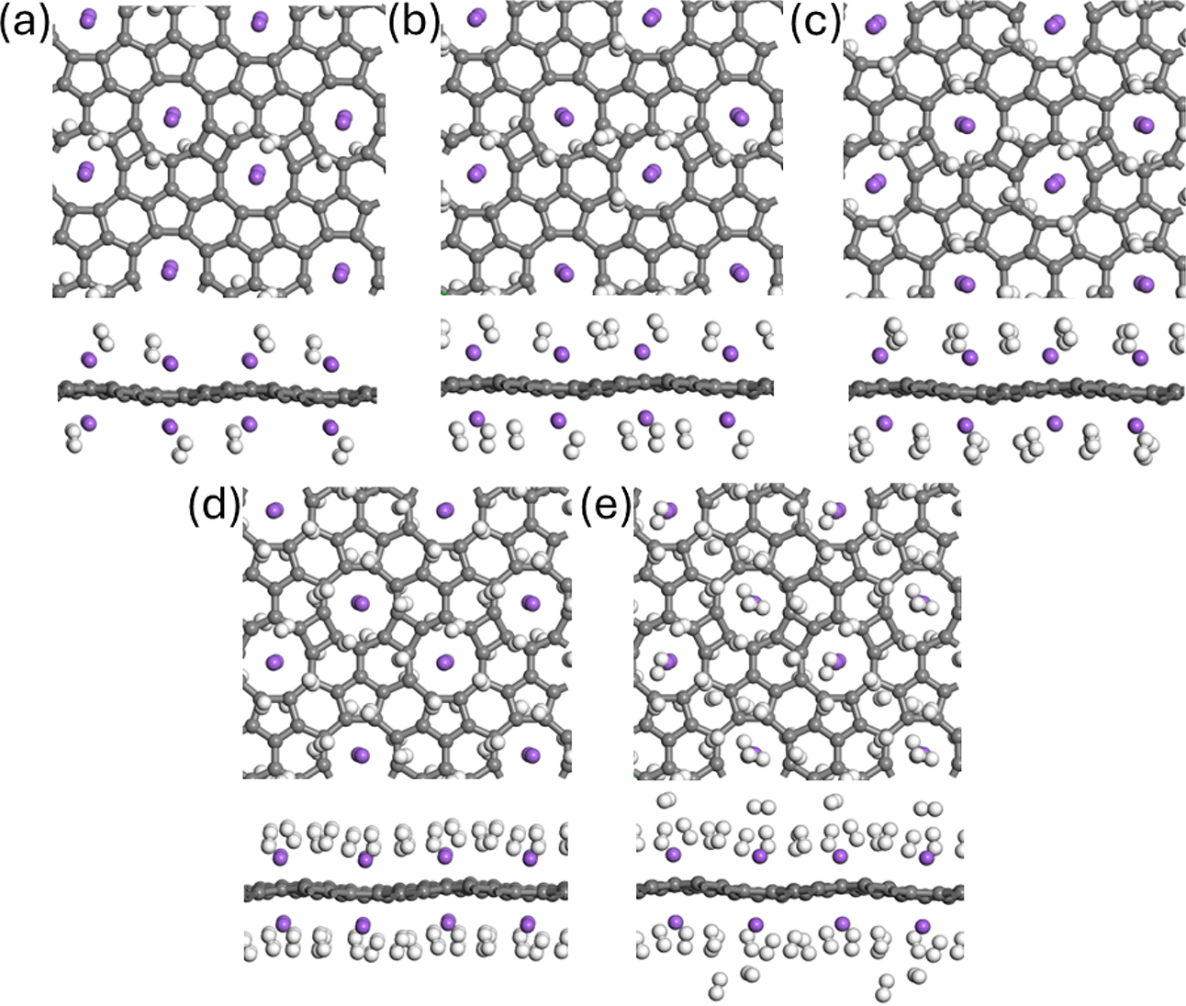
H_2_ saturation on Na@TPHE-graphene pathway, where (a), (b), (c), (d), and (e) denote Na@TPHE-graphene +4H_2_, Na@TPHE-graphene +8H_2_, Na@TPHE-graphene +12H_2_, Na@TPHE-graphene +16H_2_, and Na@TPHE-graphene +20H_2_ systems, respectively. Stepwise adsorption of 4–20 H_2_ molecules (up to 5 per Na) reveals the gradual saturation behavior of the TPHE-graphene unit cell.

Key parameters for each adsorption stage—including the average adsorption energy (*E*_ads_), consecutive adsorption energy (*E*_con_), hydrogen adsorption capacity (HAC), average H–H bond length (*R*_H–H_), and estimated desorption temperature (*T*_des_), are summarized in Table 3.

**Table 3:** Adsorption Energy (*E*_ads_), Hydrogen Adsorption Capacity (HAC), Consecutive Adsorption Energy (*E*_con_), Average H–H Bond Length (*R*_H–H_), and Desorption Temperature (*T*_des_) for Na@TPHE-Graphene + *n*H_2_ (*n* = 2, 4, 6, 8)

system	*E*_ads_ (eV)	*E*_con_ (eV)	HAC (wt %)	*R*_H–H_ (Å)	*T*_des_ (K)
Na@TPHE + 4H_2_	–0.23	–0.23	2.06	0.76	292.17
Na@TPHE + 8H_2_	–0.23	–0.26	4.04	0.77	312.41
Na@TPHE + 12H_2_	–0.23	–0.24	5.94	0.77	310.65
Na@TPHE + 16H_2_	–0.21	–0.15	7.77	0.76	280.81
Na@TPHE + 20H_2_	–0.18	–0.07	9.52	0.76	243.47

The *E*_ads_ remains nearly constant at approximately −0.23 eV up to the 12H_2_ configuration, indicating favorable and stable interactions during the initial stages of hydrogen adsorption. Beyond this point, a gradual decline in adsorption strength is observed, with *E*_ads_ decreasing to −0.21 eV for the 16H_2_ configuration and reaching −0.18 eV at full saturation (20H_2_). This trend suggests that the physisorption mechanism becomes progressively weaker as the system approaches its limit of hydrogen storage capacity.

Further insight into the energetic cost of incorporating additional H_2_ molecules is provided by the *E*_con_. This parameter reaches a peak value of −0.26 eV for the 8H_2_ configuration, followed by a gradual decline to −0.07 eV at full coverage. Such behavior reflects the progressive saturation of active sites and the increasing repulsive interactions among neighboring adsorbed molecules.

Regarding the hydrogen uptake performance, the HAC increases linearly with coverage, achieving a maximum of 9.52 wt % at full saturation, surpassing the U.S. DOE’s target for practical storage materials. Throughout all configurations, the *R*_H–H_ remains close to that of a free H_2_ molecule (0.75 Å), fluctuating only slightly between 0.76 and 0.77 Å. These values reinforce the physisorption nature of the interaction.

As for thermal behavior, the *T*_des_ remains within a practical range of 243–312 K, enabling hydrogen release under near-ambient conditions. The highest *T*_des_ of 312 K is observed for the 8H_2_ configuration, which coincides with the strongest *E*_con_ value.

To examine the interaction between H_2_ molecules with the substrate, we analyzed the CDD map, as illustrated in Figure 11. The top (Figure 11a) and side (Figure 11b) views of the CDD map reveal that adsorption is primarily driven by polarization effects, with distinct charge accumulation and depletion regions appearing on each hydrogen atom. This redistribution indicates that H_2_ molecules develop induced dipoles in the presence of the substrate, characteristic of physisorption.

**Figure 11:**
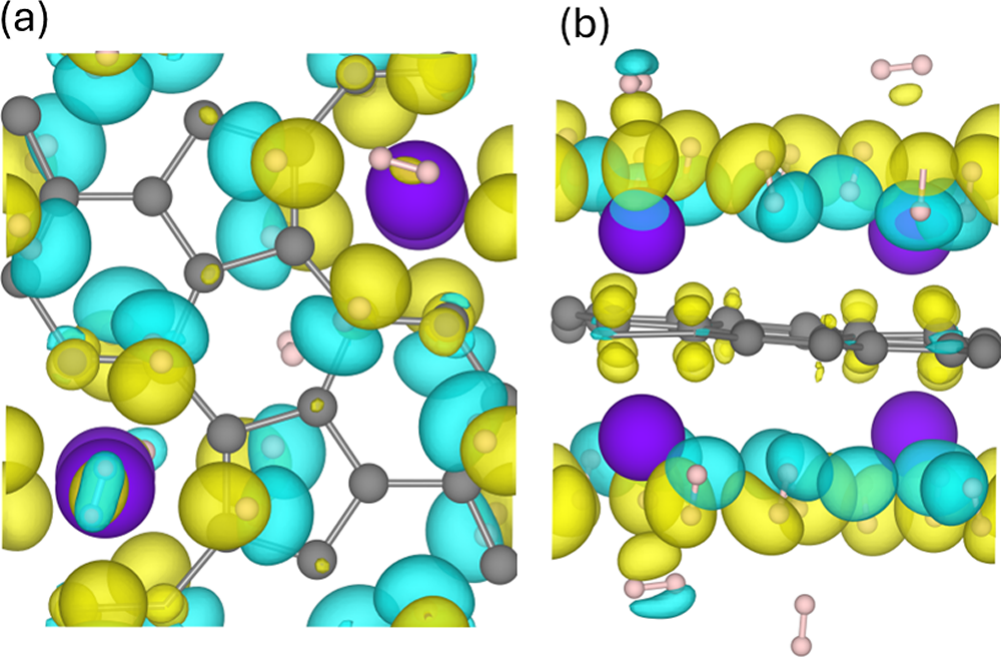
(a) Top and (b) side views of the CDD map for Na@TPHE-graphene +20H_2_. The yellow (blue) regions indicate charge accumulation (depletion).

The observed behavior aligns well with the computed adsorption energies (*E*_ads_), which fall within the physisorption regime. To further validate this mechanism, a Bader charges were obtained, revealing a net charge transfer of approximately +0.34 |*e*| for the entire Na@TPHE-graphene system and only −0.02 |*e*| per H_2_ molecule. These results confirm that the interaction involves minimal electron transfer, reinforcing the conclusion that induced dipole interactions dominate the H_2_ adsorption process.

Table 2 presents the Bader charge analysis for the Na@TPHE-graphene system under varying hydrogen loadings. As the number of adsorbed H_2_ molecules increases, a progressive charge redistribution is observed. The Na atoms consistently retain a high positive charge (approximately +3.33 |*e*|), highlighting their role as electron donors. Simultaneously, the TPHE-graphene remains with a negative charge, exhibiting slight variations (closer to −2.97 |*e*|). An accumulation of negative charge on the H_2_ molecules is noted up to 16 adsorbed H_2_, after which a slight decrease is observed at 20 H_2_, possibly due to intermolecular repulsion effects at higher coverages. This reduction may be associated with saturation effects or increased H_2_–H_2_ repulsion at high surface densities, limiting further charge accumulation per molecule.

To evaluate the thermal stability of the Na@TPHE-graphene system in the presence of hydrogen, MD simulations were conducted for the Na@TPHE-graphene +20H_2_ configuration at 300 K throughout 5 ps, as illustrated in Figure 12. The potential energy profile displays several abrupt fluctuations throughout the simulation, which are attributed to the desorption of H_2_ molecules. These events provide direct evidence of reversible hydrogen storage, with release occurring under near-ambient conditions.

**Figure 12:**
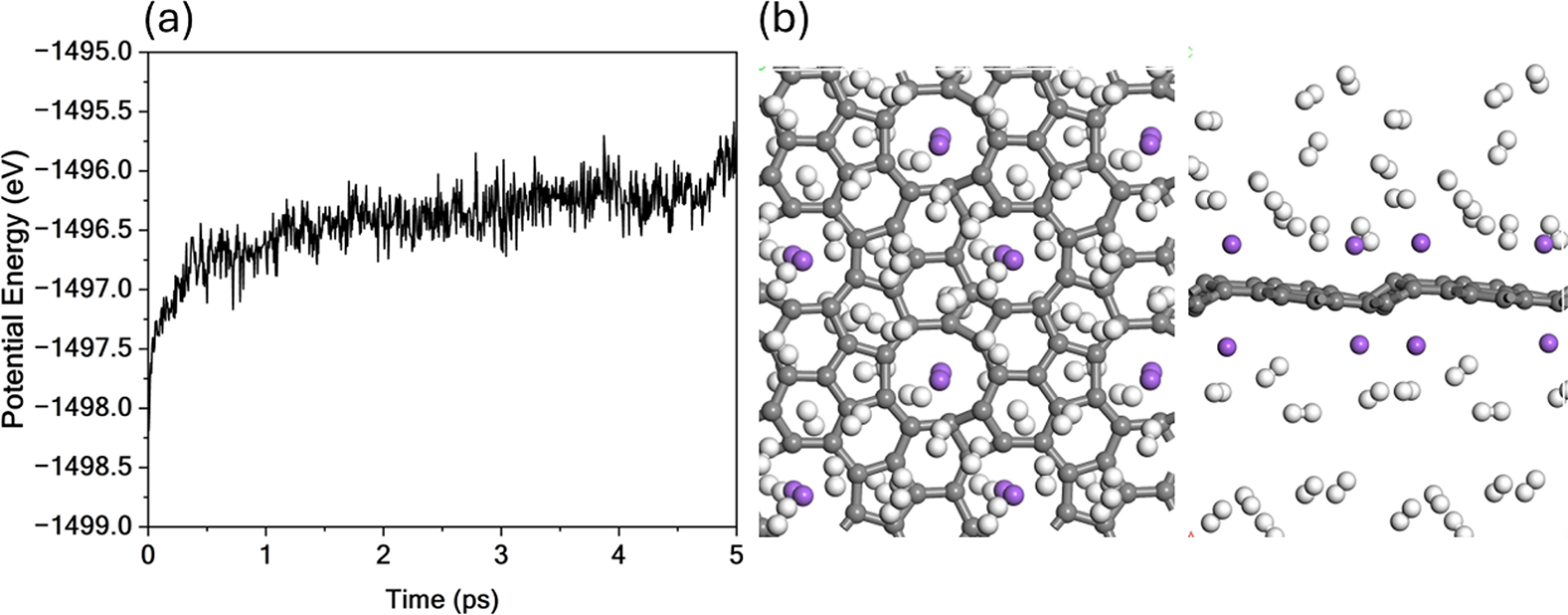
MD simulation results for Na@TPHE-graphene +20H_2_ system at 300 K. (a) Time evolution of the potential energy and (b) final system configuration for Na@TPHE-graphene +20H_2_ system. High-energy fluctuations indicate H_2_ desorption events, demonstrating the reversible storage capability of Na@TPHE-graphene under near-ambient conditions.

Despite the dynamic nature of hydrogen desorption, the substrate’s structural integrity remains intact throughout the simulation. No atomic rearrangement or bond breaking is observed, and the Na adatoms remain firmly anchored at their preferred adsorption sites, exhibiting only minimal displacements. These findings confirm that the Na-decorated TPHE-graphene maintains its structural robustness while enabling thermally activated hydrogen release.

Given the comparison with another relevant 2D system for hydrogen storage as stated in Table 4, Na@TPHE-graphene emerges as a promising candidate among recently proposed hydrogen storage systems due to its optimal combination of adsorption capacity, binding energy, and desorption temperature. In its fully saturated state with 20 adsorbed H_2_ molecules, the system shows a HAC of 9.52 wt %, outperforming several benchmark materials such as Na@B_7_N_5_ (7.70 wt %), Na@Irida-graphene (7.82 wt %), and Na@Graphdiyne (7.7 wt %).

**Table 4:** Number of Adsorbed H_2_ Molecules (*n*), Absolute Adsorption Energy per H_2_ (|*E*_ads_|), Hydrogen Adsorption Capacity (HAC), and Desorption Temperature (*T*_des_) Associated with Configurations Exhibiting Complete H_2_ Coverage Configurations in Recently Documented Systems

system	*n*	|*E*_ads_| (eV)	HAC (wt %)	*T*_des_ (K)
Na@TPHE-graphene (this work)	20	0.18	9.52	243
Na@B_7_N_5_ ^53^	32	0.20	7.70	257
Na@Irida-graphene^54^	32	0.14	7.82	195
Na@Graphdiyne^55^	5	0.25	7.7	
Na@IGP-SiC^56^	48	0.10	6.78	148
K@BP-Biphenylene^57^	32	0.14	8.27	
Mg@Me-C_8_B_5_ ^58^	3	0.29	5.74	370
NLi_4_@Phosphorene^59^	30	0.11	6.8	82
Ca@DTT-graphene^60^	5	0.22	11.7	284
Li@POG-B_4_C_2_N_3_ ^31^	10	0.19	8.35	245
Li@β_12_-borophene^61^	4	0.10	11.59	

Compared to other high-performing systems, including K@BP-Biphenylene (8.27 wt %) and Li@POG-B_4_C_2_N_3_ (8.35 wt %), Na@TPHE-graphene still exhibits superior storage capacity. Although materials like Ca@DTT-graphene (11.7 wt %) and Li@β_12_-borophene (11.59 wt %) demonstrate higher capacities, Na@TPHE-graphene balances high capacity with reversible adsorption behavior under near-ambient conditions, reinforcing its potential as a viable hydrogen storage platform.

Regarding adsorption energetics, Na@TPHE-graphene exhibits a binding energy of 0.18 eV per H_2_, which falls within the optimal range (0.15–0.25 eV) for physisorption-based hydrogen storage systems. This energy is sufficiently high to stabilize hydrogen molecules under moderate pressure yet low enough to allow for desorption without excessive thermal input.

The value is comparable to those reported for Na@B_7_N_5_ (0.20 eV) and Li@POG-B_4_C_2_N_3_ (0.19 eV) and significantly higher than that of Na@IGP-SiC (0.10 eV) and NLi_4_@Phosphorene (0.11 eV), which may exhibit insufficient binding strength for ambient-condition storage. Although systems like Mg@Me-C_8_B_5_ show stronger adsorption (0.29 eV), their practical use is limited by a lower HAC (5.74 wt %) and elevated desorption temperature (370 K). These comparisons highlight the favorable balance achieved by Na@TPHE-graphene between adsorption strength and reversibility, key attributes for viable hydrogen storage materials.

Another key parameter, the desorption temperature (*T*_des_) of 243 K, positions Na@TPHE-graphene among the most balanced candidates for practical hydrogen storage. This temperature is low enough to allow hydrogen release under near-ambient or mildly elevated conditions, yet sufficiently high to prevent premature desorption during storage.

In comparison, materials such as Na@Irida-graphene (*T*_des_ = 195 K) and NLi_4_@Phosphorene (*T*_des_ = 82 K) may suffer from hydrogen loss under mild conditions, limiting their applicability. Conversely, while systems like Mg@Me-C_8_B_5_ (370 K) and Ca@DTT-graphene (284 K) offer strong thermal stability, they require substantial heating to initiate hydrogen release, which can reduce overall energy efficiency.

To assess the electronic impact of H_2_ adsorption on Na@TPHE-graphene, we examined the electronic band structure and PDOS for the Na@TPHE-graphene +20H_2_ system, as shown in Figure 13. In the valence band, the overall band dispersion remain largely unchanged compared to both the pristine and Na-decorated TPHE-graphene cases.

**Figure 13:**
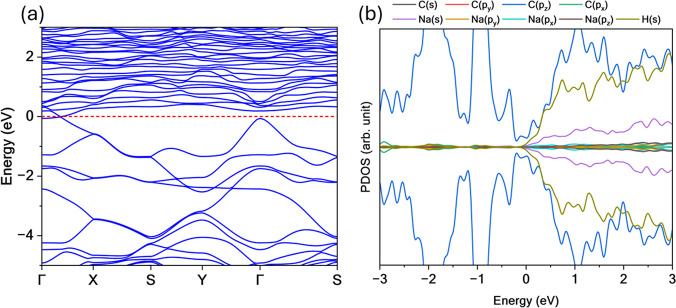
(a) Band structure and (b) PDOS for Na@TPHE-graphene +20H_2_. While the valence band retains features of the pristine and Na-decorated systems, the conduction band displays new bands and increased PDOS, mainly due to H_2_ and Na­(*s*) states. A tilted cone-like crossing at the Fermi level (red dashed line), along with reduced DOS, shows a metallic to semimetallic transition.

This observation is corroborated by the PDOS analysis, which shows negligible contributions from both Na and H_2_ in the valence band region. As with the undeformed system, the valence states are predominantly composed of carbon *p*_*z*_ orbitals, highlighting the preservation of the π-bonding network upon hydrogen adsorption.

In contrast, the conduction band exhibits noticeable modifications upon H_2_ adsorption. Several new bands emerge, accompanied by a marked increase in the density of states. The PDOS reveals that H_2_ molecules make significant contributions to the electronic states in this region, followed by substantial contributions from Na *s* orbitals.

Carbon *p*_*z*_ orbitals remain dominant in the conduction band, underscoring their continued relevance to the overall electronic structure. These findings suggest that, while the valence band remains unaffected mainly, the conduction band is more sensitive to hydrogen adsorption, potentially influencing the material’s charge transport characteristics.

A tilted cone-like crossing is observed at the Fermi level, accompanied by a reduction in the density of states at this energy. This feature indicates a transition from metallic to semimetallic behavior after full H_2_ coverage.

When analyzing the behavior of hydrogen molecules, it is essential to consider that H_2_ can be efficiently stored under low temperature and high-pressure conditions, while desorption typically occurs at elevated and reduced pressures. We refer to the thermodynamic analysis presented in Figure 14 to evaluate the practical feasibility of hydrogen adsorption and release.

**Figure 14:**
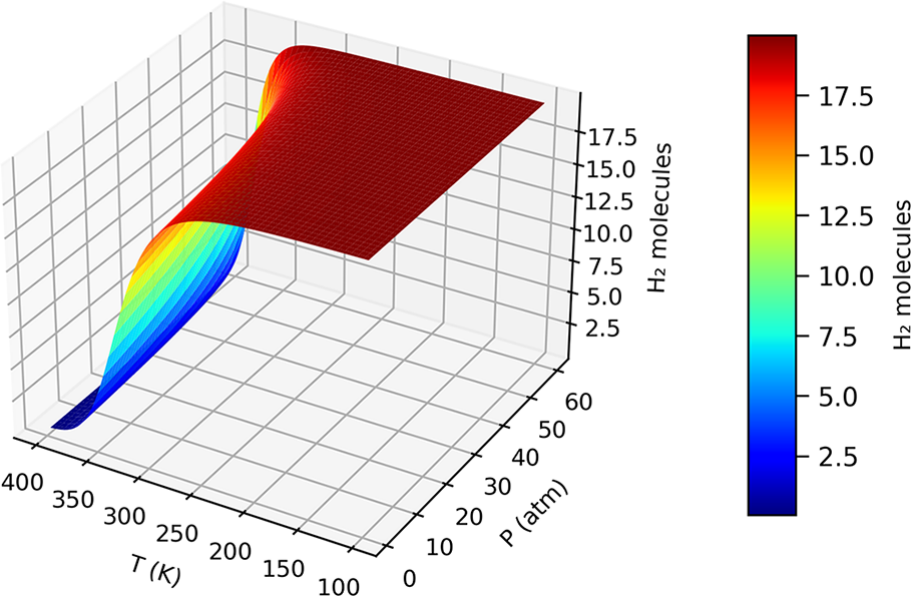
Average number of adsorbed H_2_ on Na@TPHE-graphene at various temperatures (*T*) and pressures (*P*).

In typical operating conditions, adsorption takes place at 30 atm and a temperature of 25 °C, while desorption is triggered by reducing the pressure to 3 atm and raising the temperature to 100 °C. Under these conditions, the number of adsorbed H_2_ molecules reaches 19.53 during adsorption and drops to 0.16 upon desorption. This difference corresponds to an effective hydrogen storage capacity of 9.25 wt %, demonstrating the material’s practical viability for reversible hydrogen storage applications.

In addition to the gravimetric hydrogen storage capacity (9.25 wt %), we estimate the volumetric hydrogen density of the Na@TPHE-graphene system to be approximately 52.4 g H_2_/L. This estimate assumes an interlayer spacing of 3.4 Å, commonly used for 2D materials in stacked configurations. This value exceeds the U.S. DOE 2025 target for volumetric capacity of 40 g H_2_/L.^33^

## Conclusions

In summary, we propose TPHE-graphene as a high-performance platform for hydrogen storage via sodium decoration. This novel 2D carbon monolayer, composed of 4-, 5-, 6-, and 9-membered rings, was recently identified through a high-throughput structure search. Its dynamical, energetic, and thermal stability were confirmed through phonon dispersion analysis, cohesive energy calculations, and molecular dynamics (MD) simulations.

Na decoration leads to strong chemisorption, characterized by a binding energy of −2.08 eV and significant charge transfer to the monolayer. The electronic structure remains metallic after decoration, and MD simulations confirm that Na atoms remain stably anchored at room temperature. These features ensure the structural robustness of the decorated system under realistic conditions.

Hydrogen adsorption studies reveal that each Na atom can host up to five H_2_ molecules, resulting in a gravimetric storage capacity of 9.52 wt %. Adsorption energies lie within the optimal range for physisorption, and thermodynamic simulations indicate reversible H_2_ release near ambient conditions. A practical hydrogen storage capacity of 9.25 wt % is achieved under typical working conditions, positioning Na@TPHE-graphene as a competitive and reliable candidate for solid-state hydrogen storage applications.

## Data Availability

All data supporting the findings of this study are available within the article.
